# PHYSICAL AND MENTAL HEALTH NEEDS OF OLDER ADULTS EXPERIENCING HOMELESSNESS: CHALLENGES AND INNOVATIONS IN CARE

**DOI:** 10.1093/geroni/igae098.0395

**Published:** 2024-12-31

**Authors:** Anita Souza, Jenny Tsai

**Affiliations:** Chair; Discussant

## Abstract

Adults over the age of 50 are the largest growing demographic of individuals experiencing homelessness. They enter a continuum of housing services with a myriad of chronic and acute health conditions that have often been unaddressed due to sporadic and episodic care utilization primarily within emergency services. The compounding health impacts of chronic homelessness accelerate aging within this demographic. Housing services offer an opportunity to address unmitigated health issues. Housing services are seeking research and programmatic interventions to meet this need. This symposium will explore the physical and mental health needs of older adults experiencing homelessness across the continuum of housing services from shelter entry to permanent supportive housing though a variety of research methodologies. The first presenter (Souza) will present results of a study applying latent class analysis to administrative shelter services data to identify unobservable subgroups within the population of older adults living in shelter. The second presenter (Berg) will discuss the implementation of shelter-based care services to older adults with complex medical needs in a low barrier, harm reduction focused hotel shelter. The third presenter (Robinson) will describe a qualitative investigation examining barriers to mental health care for older adults in permanent supportive housing. The discussant (Tsai) will lead symposium attendees in a discussion of future considerations for research, policy, and practice to meet the needs of this growing demographic of older adults.

